# Resolution of five-component mixture using mean centering ratio and inverse least squares chemometrics

**DOI:** 10.1186/1752-153X-7-152

**Published:** 2013-09-12

**Authors:** Mahmoud Mohamed Issa, R’afat Mahmoud Nejem, Alaa Mohamed Abu Shanab, Nahed Talab Shaat

**Affiliations:** 1 Pharmaceutical Analytical Chemistry, Department of Chemistry, Alaqsa University, P.O.Box 4051, Gaza, Palestine; 2 Analytical Chemistry, Department of Chemistry, Alaqsa University, P.O.Box 4051, Gaza, Palestine; 3 Inorganic Analytical Chemistry, Department of Chemistry, Alaqsa University, P.O.Box 4051, Gaza, Palestine; 4 R and D Department, Middle East Pharmaceuticals and Cosmetics Laboratories, Gaza, Palestine

**Keywords:** Methylparaben, Propylparaben, Chlorpheniramine, Pseudoephedrine, Mean centering ratio, Inverse least square, Spectroscopy

## Abstract

**Background:**

A comparative study of the use of mean centering of ratio spectra and inverse least squares for the resolution of paracetamol, methylparaben, propylparaben, chlorpheniramine maleate and pseudoephedrine hydrochloride has been achieved showing that the two chemometric methods provide a good example of the high resolving power of these techniques. Method (I) is the mean centering of ratio spectra which depends on using the mean centered ratio spectra in four successive steps that eliminates the derivative steps and therefore the signal to noise ratio is improved. The absorption spectra of prepared solutions were measured in the range of 220–280 nm. Method (II) is based on the inverse least squares that depend on updating developed multivariate calibration model. The absorption spectra of the prepared mixtures in the range 230–270 nm were recorded.

**Results:**

The linear concentration ranges were 0–25.6, 0–15.0, 0–15.0, 0–45.0 and 0–100.0 μg mL^-1^ for paracetamol, methylparaben, propylparaben, chlorpheniramine maleate and pseudoephedrine hydrochloride, respectively. The mean recoveries for simultaneous determination were between 99.9-101.3% for the two methods. The two developed methods have been successfully used for prediction of five-component mixture in Decamol Flu syrup with good selectivity, high sensitivity and extremely low detection limit.

**Conclusion:**

No published method has been reported for simultaneous determination of the five components of this mixture so that the results of the mean centering of ratio spectra method were compared with those of the proposed inverse least squares method. Statistical comparison was performed using t-test and F-ratio at P = 0.05. There was no significant difference between the results.

## Background

Paracetamol (PA) is an analgesic and antipyretic agent [1], which is associated with pseudoephedrine hydrochloride (PS), a direct- and indirect-acting sympathomimetic agent [2] and chlorpheniramine maleate (CH), a potent antihistaminic [3], in addition to methylparaben (MP) and propylparaben (PP), which are used as preservatives. The combination of PA, CH and PS is used for symptomatic treatment of coughs and the common cold. The UV absorption spectra of PA, MP, PP, CH, and PS displays considerable overlapping, where the application of the conventional spectrophotometry failed to resolve it. No spectrophotmetric analytical method has been reported for the simultaneous determination of PA, MP, PP, CH, and PS in a multicomponent mixture.

While official methods have been reported for the determination of each of these drugs alone in their formulations [4], the most prominent method for simultaneous determination of PA, MP, PP, CH, and PS is the HPLC, GC-MS or LC-MS [5-11]. However, these reported methods suffered from using solvents of high cost, time-consuming extraction procedure and long chromatographic retention time. In addition, the United States pharmacopeia suggested the reduction in amount of toxic organic solvents which used in HPLC assays that caused harm to human health and environment [12]. Therefore, chemometric-assisted spectrophotometry as a simple, rapid and low cost method can be a good alternative one if it is combined with multivariate calibration methods for determination of a complex in pharmaceutical quality control laboratories. The development of chemometric techniques has enabled the application to the analysis of complex mixtures without the need for any prior separation.

In particular, mean centering of ratio spectra (MCR) is used to remove the contribution of absorbing reagent from data matrix precisely and therefore the absorbance of reagent(s) is exactly eliminated [13-15]. Mean centering of ratio spectra have been presented by Afkhami and Bahram [16] applied for simultaneous analysis of binary and ternary mixture [17-22] MCR method has the advantage of eliminating the derivative steps and therefor the signal-to-noise is not degraded.

Multivariate calibration technique is gaining publicity for quantification of multi-component system even in the presence of intense spectral overlap between analytes [23,24]. Classical least squares, partial least squares, principal component regression and inverse least squares are the most common multivariate calibration tool due to their powerful calibration and ease of implementation [25-31].

In the present work a simple, rapid and inexpensive mean centering of ratio spectra (MCR) and inverse least squares methods (ILS) are developed for the resolution of five-component mixture. The results of the two chemometric assisted spectrophotometric methods were compared with each other.

### Theoretical background

#### MCR developed method

If a mixture of five compounds (PA, MP, PP, CH and PS) is considered where Beer’s law is obeyed for all compounds over the whole wavelength range used, then

(1) $$A_{m}=\propto_{\mathrm{PA}}C_{\mathrm{PA}}+\propto_{\mathrm{MP}}C_{\mathrm{MP}}+\propto_{\mathrm{PP}}C_{\mathrm{PP}}+\propto_{\mathrm{PS}}C_{\mathrm{PS}}$$

where

• A_m_ is the vector of the absorbance of the mixture,

• α_PA_, α_MP_, α_PP_, α_CH_, and α_PS_ are the absorptivity vectors of PA, MP, PP, CH

• and PS and C_PA_, C_MP_, C_PP_, C_CH_, and C_PS_ are the concentrations of PA, MP, PP, CH and PS, respectively.

If Equation (1) is divided by α_CH_ corresponding to the spectrum of a standard solution of CH in the mixture, the first ratio spectrum is obtained

(2) $$\begin{array}{ll} \;x=\frac{A_{m}}{\propto_{\mathrm{CH}}}= & \frac{\propto_{\mathrm{PA}}C_{\mathrm{PA}}}{\propto_{\mathrm{CH}}}+\frac{\propto_{\mathrm{MP}}C_{\mathrm{MP}}}{\propto_{\mathrm{CH}}}+\frac{\propto_{\mathrm{PP}}C_{\mathrm{PP}}}{\propto_{\mathrm{CH}}}+C_{\mathrm{CH}}\\ & +\frac{\propto_{\mathrm{PS}}C_{\mathrm{PS}}}{\propto_{\mathrm{CH}}} \end{array}$$

If the Equation (2) is mean centered, then

(3) $$\begin{array}{ll} \;\mathrm{mc}x= & \mathrm{mc}\frac{\propto_{\mathrm{PA}}C_{\mathrm{PA}}}{\propto_{\mathrm{CH}}}+\mathrm{mc}\frac{\propto_{\mathrm{MP}}C_{\mathrm{MP}}}{\propto_{\mathrm{CH}}}+\mathrm{mc}\frac{\propto_{\mathrm{PP}}C_{\mathrm{PP}}}{\propto_{\mathrm{CH}}}\\ & +\frac{\propto_{\mathrm{PS}}C_{\mathrm{PS}}}{\propto_{\mathrm{CH}}} \end{array}$$

and if Equation (3) is divided by $\mathrm{mc}\frac{\alpha_{\mathrm{PA}}}{\alpha_{\mathrm{CH}}}$, the second ratio spectrum is obtained

$$y=\frac{\mathrm{mc}\;x}{\mathrm{mc}\frac{\;\alpha_{\mathrm{PA}}}{\alpha_{\mathrm{CH}}}}=C_{\mathrm{PA}}+Z\;C_{\mathrm{MP}}+\;\frac{\mathrm{mc}\frac{\alpha_{\mathrm{PP}}C_{\mathrm{PP}}}{\alpha_{\mathrm{CH}}}}{\mathrm{mc}\frac{\;\alpha_{\mathrm{PA}}}{\alpha_{\mathrm{CH}}}}\;+\;\frac{\mathrm{mc}\frac{\alpha_{\mathrm{PS}}C_{\mathrm{PS}}}{\alpha_{\mathrm{CH}}}}{\mathrm{mc}\frac{\;\alpha_{\mathrm{PA}}}{\alpha_{\mathrm{CH}}}}$$

(4) $$\mathrm{mc}\;y\;=\;\mathrm{mc}\;Z\;C_{\mathrm{MP}}+\mathrm{mc}\;\frac{\mathrm{mc}\frac{\alpha_{\mathrm{PP}}}{\alpha_{\mathrm{CH}}}C_{\mathrm{PP}}}{\mathrm{mc}\frac{\;\alpha_{\mathrm{PA}}}{\alpha_{\mathrm{CH}}}}+\;\mathrm{mc}\;\frac{\mathrm{mc}\frac{\alpha_{\mathrm{PS}}}{\alpha_{\mathrm{CH}}}C_{\mathrm{PS}}}{\mathrm{mc}\frac{\;\alpha_{\mathrm{PA}}}{\alpha_{\mathrm{CH}}}}$$

In the same way, the third ratio spectrum can be obtained

(5) $$\mathrm{mc}\;\frac{\mathrm{mc}\;y}{\mathrm{mc}\;z}=mc_{o}C_{\mathrm{PP}}+\frac{\mathrm{mc}\frac{\mathrm{mc}\;\propto_{\mathrm{PP}}/\propto_{\mathrm{CH}\;}}{\mathrm{mc}\;\propto_{\mathrm{PA}}/\propto_{\mathrm{CH}}}C_{\mathrm{PS}}}{\mathrm{mc}\;z}$$

Finally we obtain the fourth ratio spectrum

(6) $$\mathrm{mc}\;\frac{\mathrm{mc}\;y/\mathrm{mc}\;z}{mc_{o}}=KC_{\mathrm{PS}}\;\left(K\;\mathrm{is}\;a\;\mathrm{constant}\right)$$

Equation (6) is the mathematical basis of multicomponent analysis which permits the determination of the concentration of each compounds without interference from the other components of the mixture.

In practice, the signal of the fourth ratio spectrum of PS is dependent only on the concentration value C_PS_ and $\frac{A_{m}}{\alpha_{\mathrm{CH}}}$, but is independent of the concentration values C_PA_, C_MP_, C_PP_, and C_CH_ in the mixture. In the developed method, the concentration C_PS_ in the mixture is proportional to the amount of $\mathrm{mc}\;\frac{\mathrm{mc}y/\mathrm{mc}Z}{\mathrm{mc}\;o}$ corresponding to a maximum or minimum point.

A calibration curve could be constructed by plotting $\mathrm{mc}\;\frac{\mathrm{mc}y/\mathrm{mc}Z}{\mathrm{mc}\;o}$ against different concentration of PS. As explained previously, this technique can be used for other systems, particularly for more than five components system. By using the calibration curve, the concentration of PS was determined in a sample containing PA, MA, PP and CH. The concentrations of the other components (PA, MA, PP and CH) are determined separately by analogous procedures of PS.

### ILS method

The mathematical formulations of this method, in the matrix compact form, can be written as

(7) C=PA+E

where the matrix A represents the absorbance matrix, C is the concentration matrix, P is the calibration coefficients and E is a matrix of concentration prediction error. This inverse Beer’s law expression implies that the concentration is a function of the absorbance at a series of given wavelengths. The P matrix of coefficients can be solved by computing:

(8) $$P=CA^{-1}$$

if the A matrix is not square, the pseudo-inverse must be used instead :

(9) $$\hat{P}=C\;A^{\prime }\;{\left(AA^{\prime }\right)}^{-1}$$

Therefore the C matrix can be determined by using the following equation

(10) $$C=\hat{P}A$$

This model appears to be the best approach for almost all quantitative analyses since no knowledge of the sample composition is needed beyond the concentrations of the constituents of interest.

## Materials and methods

### Instrumentation and software

A shimadzu (Kyoto, Japan) UV-1650 PC, UV-Visible double-beam spectrophotometer with two matched 1 cm path-length quartz cells was used. This instrument is used for all the absorbance measurements. Using the “online matrix calculator bluebit, powered by Net Matrix Library (http://www.bluebit.gr/matrix-calculator), all the treatment of data was performed. The subsequent statistical manipulations were performed by transferring the spectral data to Microsoft Excel 2010 program and SPSS.

### Reagents and materials

Pharmaceutical grade of PA, MP, PP, CH and PS with claimed purities of 99.8, 99.9, 99.7, 99.7 and 99.9%, respectively according to manufactures certificate were kindly donated by the Middle East pharmaceuticals and cosmetics laboratories, Palestine.

Decamol Flu syrup (batch number 1943) (Middle East pharmaceuticals and cosmetics laboratories, Palestine) was used. Each 5.0 ml contains 160 mg PA, 5.0 mg MP, 1.0 mg PP, 1.0 mg CH and 1.0 mg PS.

#### Stock standard and working solutions

Stock solutions of PA, MP, PP, CH and PS were independently prepared by dissolving 100.0 mg of each in 100.0 mL of 0.1 M HCl (Merck). Working solutions were prepared by transferring appropriate volumes of the stock solutions to separate 25.0 ml volumetric flasks and diluted to their marks with 0.1 M HCl. A series of five solutions of each compound in the concentration range of 0–25.6 μg mL^-1^ for PA, 0–15.0 μg mL^-1^ MP, 0–15.0 μg mL^-1^ PP, 0–45.0 μg mL^-1^ CH and 0–100.0 μg mL^-1^ PS was obtained from the stock solutions. A 25 laboratory sample mixtures containing different ratios of the five studied components were prepared and used in the calibration and validation sets.

### Procedures

#### Mean centering of ratio spectra method (MCR)

The absorption spectra of prepared solutions were measured in the range of 220–280 nm. Beer’s law was obeyed for all compounds over the entire wavelengths (220–280 nm).

For PS, the recorded spectra were divided by standard spectrum of 1.0 μg mL^-1^ CH to obtain the first ratio spectra which was then mean centered. These vectors were then divided by the mean center of $\frac{\alpha_{\mathrm{PA}}}{\alpha_{\mathrm{CH}}}$ and therefore the mean centering of the second ratio spectra were obtained. In the same way, the third and fourth ratio spectra can be obtained as shown in Table 1.

**Table 1:** The first, second, third and fourth ratio spectra data

**Drug**	**X**	**Y**	**Z**	**O**	**Divisors used**
PS	$\frac{A_{m}}{\alpha_{\mathrm{CH}}}$	$\frac{\mathrm{mc}\;x}{\mathrm{mc}\frac{\;\alpha_{\mathrm{PA}}}{\alpha_{\mathrm{CH}}}}$	$\frac{\mathrm{mc}\frac{\alpha_{\mathrm{MP}}}{\alpha_{\mathrm{CH}}}}{\mathrm{mc}\frac{\;\alpha_{\mathrm{PA}}}{\alpha_{\mathrm{CH}}}}$	$\frac{\;\mathrm{mc}\;\frac{\mathrm{mc}\;\alpha_{\mathrm{PP}}/\alpha_{\mathrm{CH}}}{\mathrm{mc}\;\alpha_{\mathrm{PA}}/\alpha_{\mathrm{CH}}}}{\mathrm{mc}\;z}$	1.0 μg mL^-1^ CH
CH	$\frac{A_{m}}{\alpha_{\mathrm{PA}}}$	$\frac{\mathrm{mc}\;x}{\mathrm{mc}\frac{\;\alpha_{\mathrm{PP}}}{\alpha_{\mathrm{PA}}}}$	$\frac{\mathrm{mc}\frac{\alpha_{\mathrm{MP}}}{\alpha_{\mathrm{PA}}}}{\mathrm{mc}\frac{\;\alpha_{\mathrm{PP}}}{\alpha_{\mathrm{PA}}}}$	$\frac{\;\mathrm{mc}\;\frac{\mathrm{mc}\;\alpha_{\mathrm{PS}}/\alpha_{\mathrm{PA}}}{\mathrm{mc}\;\alpha_{\mathrm{PP}}/\alpha_{\mathrm{PA}}}}{\mathrm{mc}\;z}$	10.0 μg mL^-1^ PA
PP	$\frac{A_{m}}{\alpha_{\mathrm{CH}}}$	$\frac{\mathrm{mc}\;x}{\mathrm{mc}\frac{\;\alpha_{\mathrm{PA}}}{\alpha_{\mathrm{CH}}}}$	$\frac{\mathrm{mc}\frac{\alpha_{\mathrm{MP}}}{\alpha_{\mathrm{CH}}}}{\mathrm{mc}\frac{\;\alpha_{\mathrm{PA}}}{\alpha_{\mathrm{CH}}}}$	$\frac{\;\mathrm{mc}\;\frac{\mathrm{mc}\;\alpha_{\mathrm{PS}}/\alpha_{\mathrm{CH}}}{\mathrm{mc}\;\alpha_{\mathrm{PA}}/\alpha_{\mathrm{CH}}}}{\mathrm{mc}\;z}$	10.0 μg mL^-1^ CH
MP	$\frac{A_{m}}{\alpha_{\mathrm{CH}}}$	$\frac{\mathrm{mc}\;x}{\mathrm{mc}\frac{\;\alpha_{\mathrm{PA}}}{\alpha_{\mathrm{CH}}}}$	$\frac{\mathrm{mc}\frac{\alpha_{\mathrm{PP}}}{\alpha_{\mathrm{CH}}}}{\mathrm{mc}\frac{\;\alpha_{\mathrm{PA}}}{\alpha_{\mathrm{CH}}}}$	$\frac{\;\mathrm{mc}\;\frac{\mathrm{mc}\;\alpha_{\mathrm{PS}}/\alpha_{\mathrm{CH}}}{\mathrm{mc}\;\alpha_{\mathrm{PA}}/\alpha_{\mathrm{CH}}}}{\mathrm{mc}\;z}$	10.0 μg mL^-1^ CH
PA	$\frac{A_{m}}{\alpha_{\mathrm{CH}}}$	$\frac{\mathrm{mc}\;x}{\mathrm{mc}\frac{\;\alpha_{\mathrm{PS}}}{\alpha_{\mathrm{CH}}}}$	$\frac{\mathrm{mc}\frac{\alpha_{\mathrm{MP}}}{\alpha_{\mathrm{CH}}}}{\mathrm{mc}\frac{\;\alpha_{\mathrm{PS}}}{\alpha_{\mathrm{CH}}}}$	$\frac{\;\mathrm{mc}\;\frac{\mathrm{mc}\;\alpha_{\mathrm{PP}}/\alpha_{\mathrm{CH}}}{\mathrm{mc}\;\alpha_{\mathrm{PS}}/\alpha_{\mathrm{CH}}}}{\mathrm{mc}\;z}$	10.0 μg mL^-1^ CH

The mean centered values of the fourth ratio spectra at 265, 230, 230, 240 and 260 nm for PA, MP, PP, CH and PS, respectively were measured and plotted against the correspond concentration of each drug to construct their calibration curves.

Different synthetic mixtures containing different ratios of PA, MP, PP, CH and PS within their calibration ranges were prepared. The spectra of these mixtures were recorded and the MCR procedure was performed to predict the concentration of each compound in the mixture.

2.0 ml of Decamol Flu syrup was transferred to 100.0 ml volumetric flasks (five times) dissolved in 0.1 M HCl. Then 1 ml of the solution was transferred to 25.0 ml volumetric flasks and the volume was completed with the same solvent. The proposed method was applied to the prepared solutions.

#### Inverse least squares method (ILS)

Multilevel multifactor design was used for construction of calibration and validation sets. A five-level, five-factor calibration design was used in order to prepare 25 laboratory prepared mixtures containing different ratios of the five studied drugs, the concentrations details are given in Table 2. The absorption spectra of the prepared mixtures in the range 230–270 nm were recorded. The inverse Beer’s law was obeyed where the concentration is a function of the absorbance at a series of wavelengths (230, 235, 240, 245, 250, 255, 260, 265 and 270 nm). The absorbance’s data were obtained by measuring at nine points with intervals of Δλ = 5 nm in the spectrum. Seventeen mixtures were used for building the calibration model. The remaining eight mixtures were used for validation set. The concentration of each component was calculated using the calibration model. The proposed method was applied to the previously prepared solutions of Decamol Flu syrup.

**Table 2:** **Concentrations of PA, PS, MP, PP and CH (μg mL**^**-1**^**) in the calibration and validation sets**

**Sample No.**	**PA**	**PS**	**MP**	**PP**	**CH**
1	15.0	0.00	1.50	3.00	4.00
2	25.6	5.00	1.50	3.00	5.00
3	25.6	7.00	0.80	0.16	3.00
4^*^	20.0	2.40	0.80	0.16	5.00
5	20.0	0.00	4.00	5.00	5.00
6	25.6	0.00	4.00	5.00	4.00
7	10.0	10.0	1.50	3.00	3.00
8^*^	15.0	5.00	2.00	4.00	2.00
9	10.0	0.00	4.00	5.00	4.00
10	20.0	7.00	1.50	3.00	2.00
11^*^	20.0	5.00	1.00	2.00	0.16
12	5.00	10.0	2.00	4.00	4.00
13	10.0	2.40	2.00	4.00	2.00
14^*^	25.6	0.00	1.00	2.00	5.00
15^*^	15.0	7.00	1.00	2.00	4.00
16	20.0	7.00	2.00	4.00	3.00
17	5.00	2.40	1.00	2.00	3.00
18^*^	15.0	5.00	0.80	0.16	0.16
19	25.6	2.40	2.00	4.00	0.16
20^*^	5.00	10.0	1.50	3.00	5.00
21	5.00	7.00	4.00	5.00	2.00
22	10.0	2.40	1.00	2.00	4.00
23^*^	20.0	10.0	0.80	0.16	0.16
24	15.0	10.0	4.00	5.00	0.16
25	5.00	5.00	0.80	0.16	3.00

*Samples used for validation.

## Results and discussion

**Figure 1:**
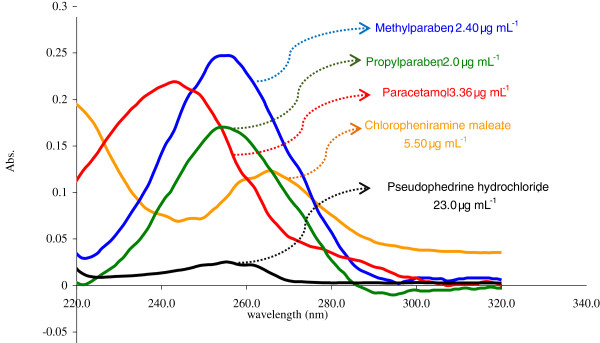
The zero order spectra of PA; MP; PP; CH; PS in 0.1 M HCl.

The absorption spectra of PA, MP, PP, CH and PS, Figure 1, displays considerable overlapping, where the application of conventional spectrophotometry failed to resolve these overlapping. To the best of our knowledge, there are no other techniques for the simultaneous spectrophotometry determination of these drugs by chemometric methods.

### Mean centering of ratio spectra method (MCR)

The developed MCR method depends on the mean centering of ratio spectra, it eliminates the derivative steps and therefore signal to noise ratio is enhanced [16] and it has been applied for resolving the five-component mixture.

In order to optimize the developed MCR method, effect of divisor on the selectivity of the method has been tested. Different concentrations of each CH, PS and MP were tested. Results in Table 1 shows that the divisor had a great effect on the selectivity of determination of PA, MP, PP, CH and PS where reproducible and good results have been obtained upon using concentration of 10.0 μg mL^-1^ of CH (for PP, MP and PA), 1.0 μg mL^-1^ CH (for PS) and 10.0 μg mL^-1^ PA (for CH) as divisors. On the other hand, changing the concentration of the divisor had no significant effect on the analytical parameters. The amount of Δλ had no effect on the mean centering of ratio spectra. A Δλ of 5 nm was used.

**Figure 2:**
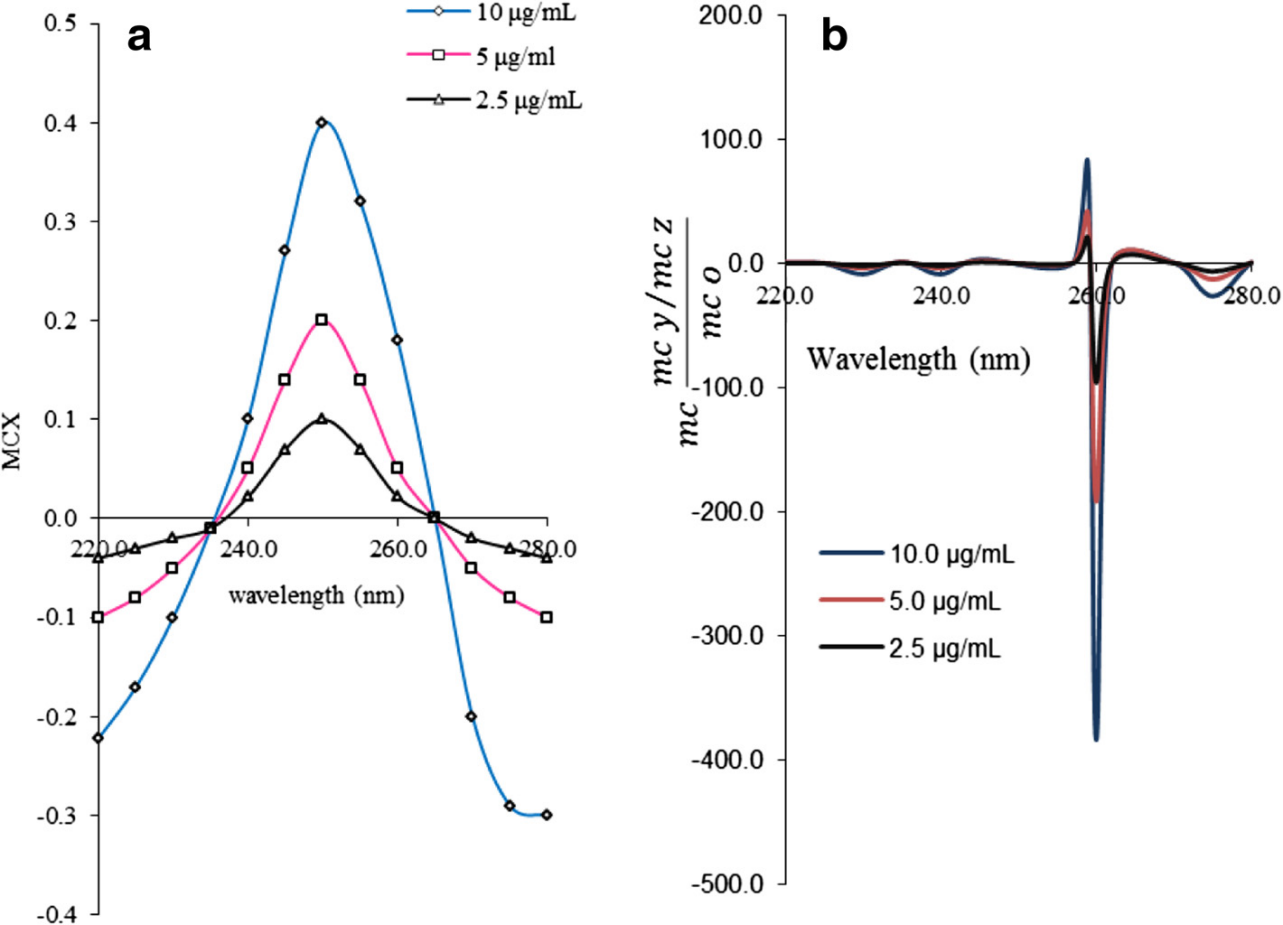
The first ratio spectra of different concentrations of PS (a) and fourth ratio spectra (b).

The absorption spectra of the standard solutions of PS was divided by the normalized spectrum of 1.0 μg mL^-1^ CH and the first ratio spectra were obtained (Figure 2a). After that the fourth ratio spectra according to Equation 6 were obtained. The concentration of PS was determined by measuring the amplitude at 260 nm corresponding to a minimum wavelength in the fourth ratio spectra as shown in (Figure 2b). For the prediction of concentration of PS in synthetic mixtures and real samples, the sample was done in similar steps.

**Figure 3:**
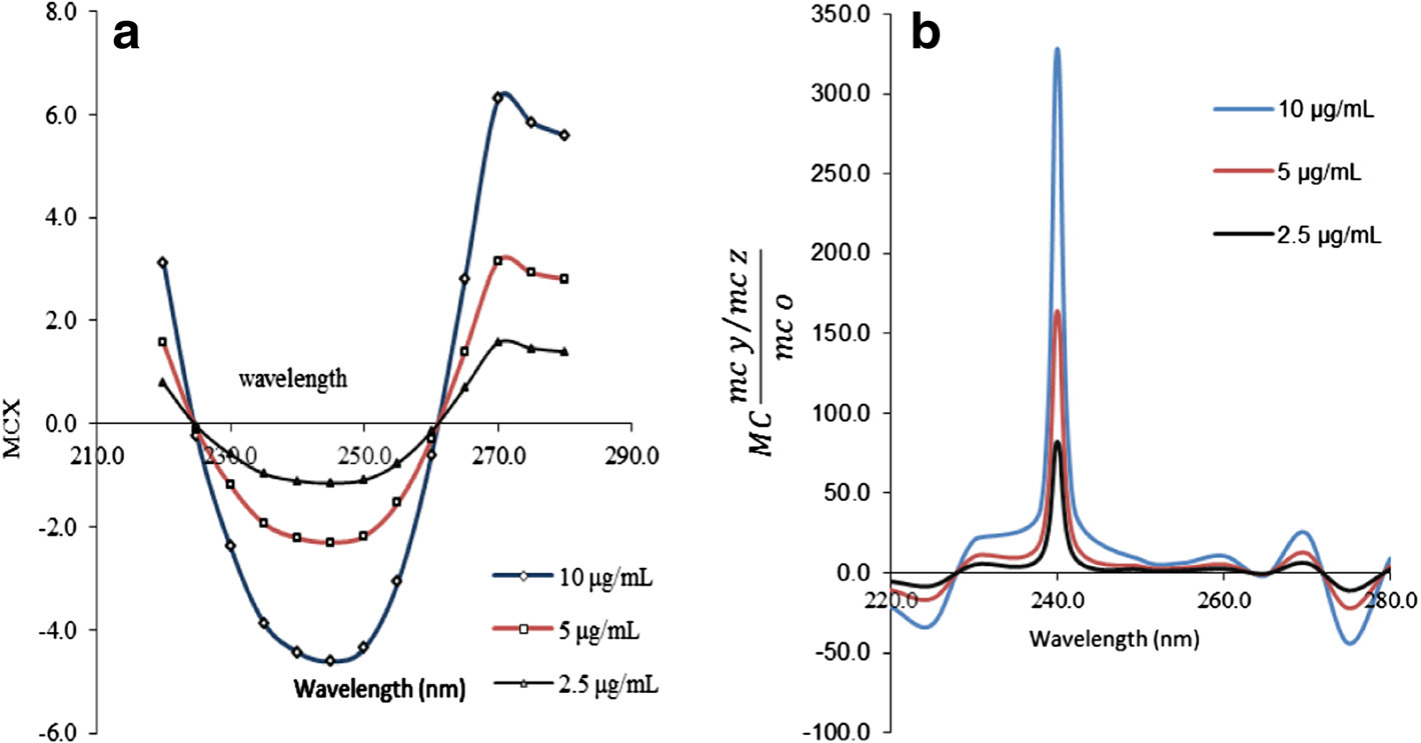
The first ratio spectra of different concentrations of CH (a) and fourth ratio spectra (b).

**Figure 4:**
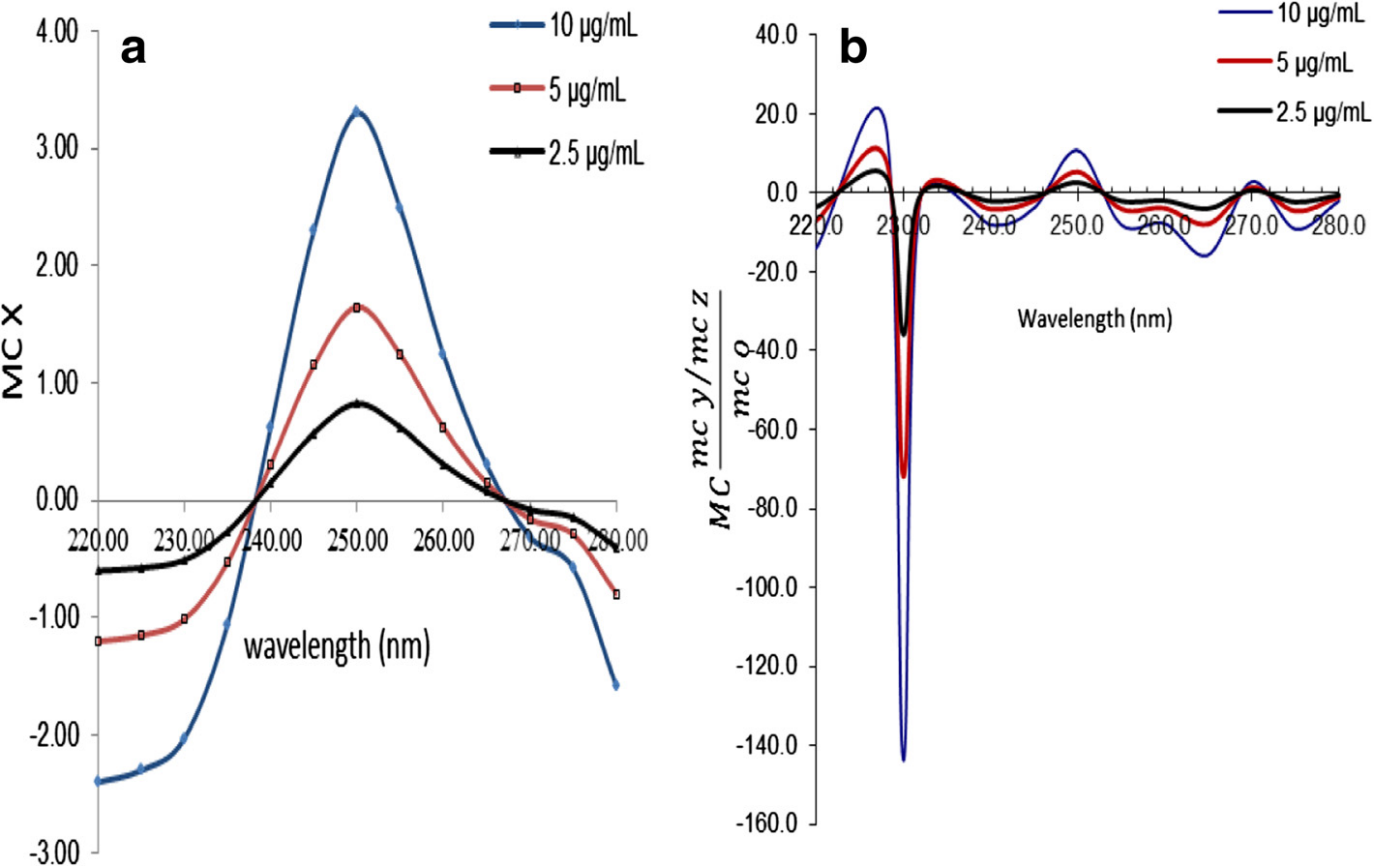
The first ratio spectra of different concentrations of PP (a) and fourth ratio spectra (b).

**Figure 5:**
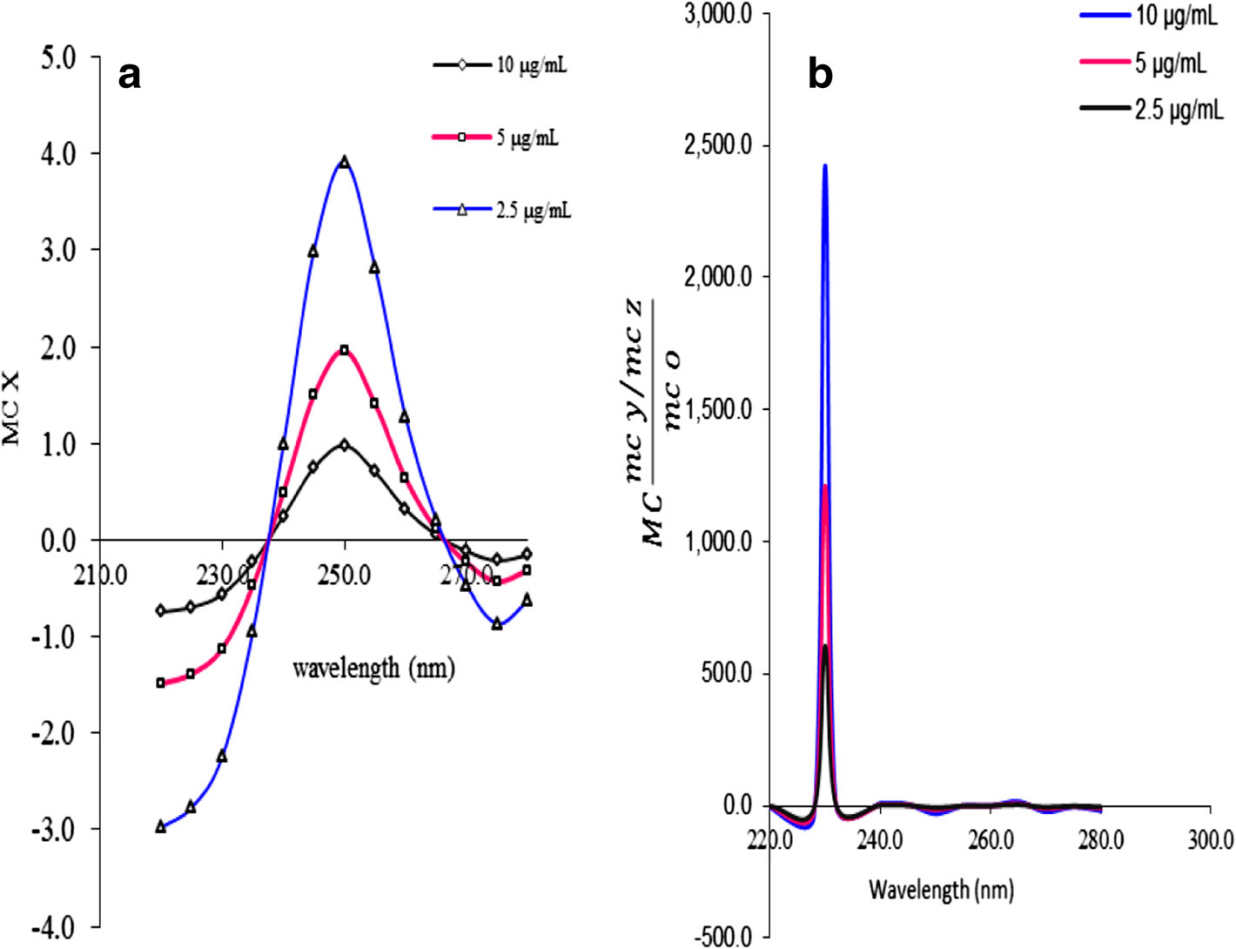
The first ratio spectra of different concentrations of MP (a) and fourth ratio spectra (b).

**Figure 6:**
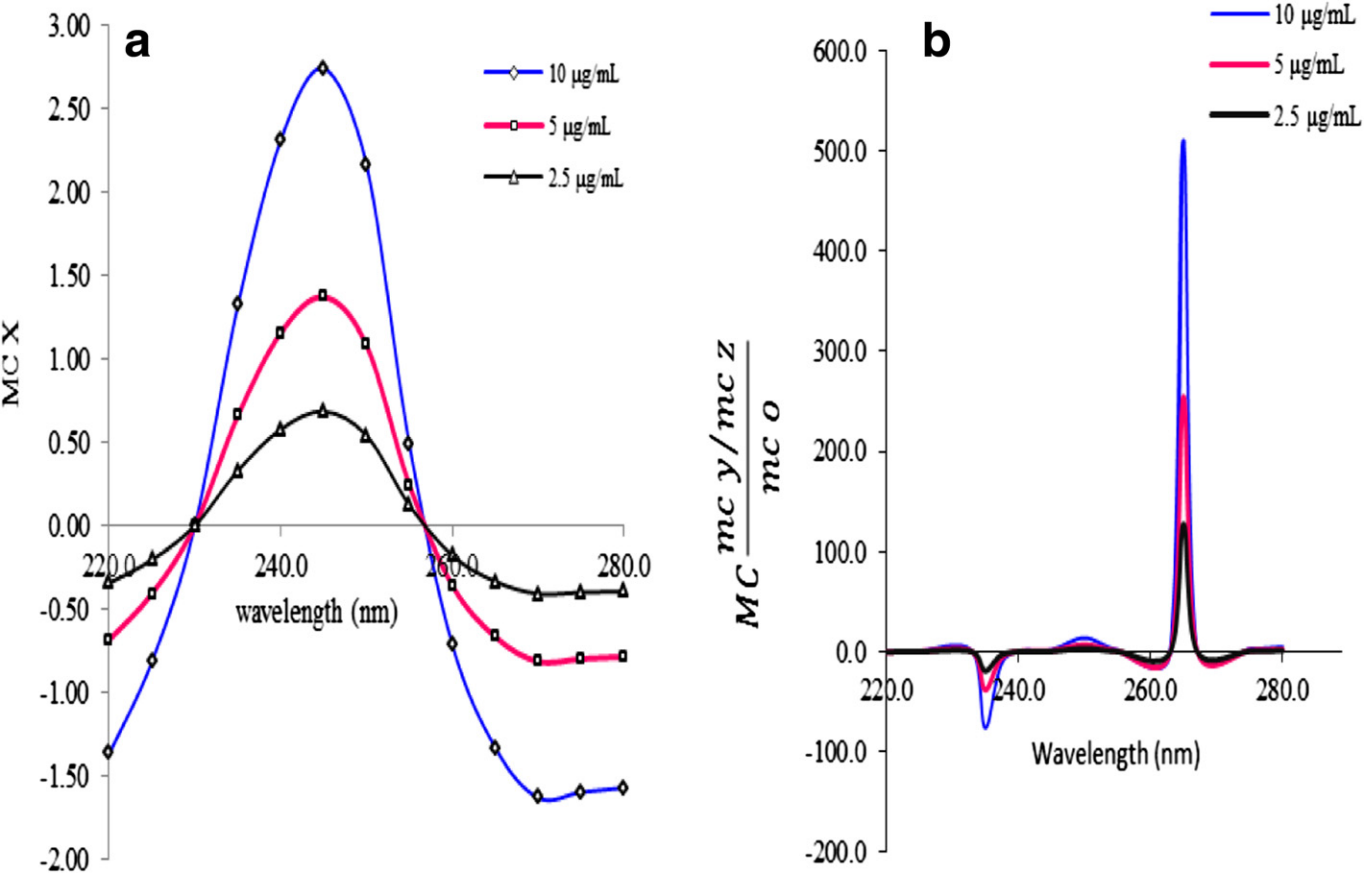
The first ratio spectra of different concentrations of PA (a) and fourth ratio spectra (b).

In the same way, the first ratio spectra for CH (Figure 3a), PP (Figure 4a), MP (Figure 5a) and PA (Figure 6a) were obtained. And the fourth ratio spectra were also obtained for other drugs. The concentration of PS, CH, PP, MP and PA was determined by measuring the amplitude at 260 (Figure 2b), 240 (Figure 3b), 230 (Figure 4b), 230 (Figure 5b) and 265 nm (Figure 6b), respectively.

Beer’s law was obeyed in the concentration 0–25.6 μg mL^-1^ for PA, 0–15.0 μg mL^-1^ for MP, 0–15.0 μg mL^-1^ PP, 0–45.0 μg mL^-1^ CH and 0–100.0 μg mL^-1^ PS. Table 3 shows the linear regression parameters for calibration data for simultaneous determination of PA, MP, PP, CH and PS in their five-component mixtures. Limit of detections (defined as the concentration equivalent to three times the standard deviation of five replicate measurements of the blank) are also shown in Table 3.

**Table 3:** Analytical characteristics for analysis of PA, PS, MP, PP and CH by MCR method

**Analyte**	**λ (nm)**	**Calibration equations**	**R**^**2**^	**Linear concentration range (μg mL**^**-1**^**)**	**LOD (μg mL**^**-1**^**)**
PA	265.0	Y = 47.28C + 2.6	0.9999	0-25.6	0.05
MP	230.0	Y = 224.6C-2.03	0.9991	0-15.0	0.05
PP	230.0	Y = −13.0C + 1.13	0.9995	0-15.0	0.05
CH	240.0	Y = 29.77C + 1.13	0.9981	0-45.0	0.08
PS	260.0	Y = −35.75C-1.25	0.9967	0-100.0	0.08

To check the reproducibility of the method, the relative standard deviation (R.S.D) for five replicate determinations of 5.0 μg mL^-1^ of each PA, MP, PP, CH and PS, in five-component mixtures were obtained as 1.91, 1.58, 1.58, 2.45 and 1.66%, respectively. The mean recoveries for simultaneous determination of the five components were obtained as 100.0, 99.9, 100.1, 101.3 and 101.1% for PA, MP, PP, CH and PS, respectively.

In order to obtain the accuracy and precision of the method, several synthetic mixtures with different concentration ratios of PA, MP, PP, CH and PS were analyzed using the proposed method. The results are summarized in Table 4. The prediction error of single component in the mixtures was calculated as the relative standard error (R.S.E) of the prediction concentration [32].

$$R.S.E\left(\%\right)={\left(\frac{\sum_{j=1}^{N}{\left({\hat{C}}_{j}-C_{j}\right)}^{2}}{\sum_{j=1}^{N}{\left(C_{j}\right)}^{2}}\right)}^{\frac{1}{2}}\;\times \;100$$

where N is the number of samples, *C*_*j*_ the concentration of component in the mixture and *Ĉ*_*j*_ the estimated concentration. The total prediction error of N samples is calculated as follows:

$$R.S.E._{t}\left(\%\right)={\left(\frac{\sum_{i=1}^{M}\sum_{j=1}^{N}{\left({\hat{C}}_{i\;j}-C_{i\;j}\right)}^{2}}{\sum_{i=1}^{M}\sum_{j=1}^{N}{\left(C_{i\;j}\right)}^{2}}\right)}^{\frac{1}{2}}\;\times \;100$$

where *C*_*ij*_ is the concentration of the component in the j^th^ samples and *Ĉ*_*ij*_ its estimation. Table 4 also includes the single and total relative errors for each the five component mixtures.

**Table 4:** Analysis of PA, PS, MP, PP and CH in synthetic mixture by MCR method

**Taken (μg mL**^**-1**^**)**					**Found (μg mL**^**-1**^**)**					**Recovery, %**				
**PA**	**MP**	**PP**	**CH**	**PS**	**PA**	**MP**	**PP**	**CH**	**PS**	**PA**	**MP**	**PP**	**CH**	**PS**
20.0	0.80	0.16	5.00	2.40	20.19	0.816	0.158	5.14	2.38	100.95	102.0	99.0	102.8	99.0
15.0	2.00	4.00	2.00	5.00	14.92	1.93	4.10	2.12	4.96	99.74	96.0	102.5	106.0	99.2
20.0	1.00	2.00	0.16	5.00	20.40	0.99	2.02	0.17	4.99	102.10	99.0	101.0	106.0	99.8
25.6	1.00	2.00	5.00	0.00	25.31	0.98	2.06	4.91	0.00	98.88	98.0	103.0	98.2	102
15.0	1.00	2.00	4.00	7.00	15.41	1.00	1.90	3.93	7.23	102.73	100.0	95.0	98.3	103.3
15.0	0.80	0.16	0.16	5.00	15.17	0.832	0.16	0.155	5.11	101.11	104.0	100.0	97.0	102.2
5.00	1.50	3.00	5.00	10.0	4.95	1.55	3.08	5.19	10.3	99.00	103.3	102.3	103.8	103.0
20.0	0.80	0.16	0.16	10.0	19.72	0.776	0.157	0.157	10.0	98.60	97.0	98.0	98.0	100.0
Mean recovery										100.39	99.91	100.10	101.26	101.06
R.S.E single (%)										1.381	3.123	2.864	2.959	2.183
R.S.E_t_ (total) (%)												1.624		

### Inverse least squares method (ILS)

As explained in the previous section, the constant (P) values were calculated by using the linear equation system between the absorbance data and calibration set. The coefficients matrix (P) was placed in the linear equation system; the following expressions for the nine wavelengths were obtained as

(11) $$\begin{array}{l} 14.{4693A}_{1}-6.{34365A}_{2}+1.240{42A}_{3}+13.5241\\ \;+0.{12545A}_{5}+0.0{49169A}_{6}\\ \;+1.{56462A}_{7}-9.0{5168A}_{8}-11.30{68A}_{9}=C_{\mathrm{PA}} \end{array}$$

(12) $$\begin{array}{l} -80.{6666A}_{1}+35.0{631A}_{2}-6.91880A_{3}\\ \;+181.{818A}_{4}-165.{446A}_{5}-0.29030{6A}_{6}-8.790{16A}_{7}\\ \;+3.{42144A}_{8}+62.{5319A}_{9}\\ \;=C_{\mathrm{MP}} \end{array}$$

(13) $$\begin{array}{l} 71.30{36A}_{1}-43.{1749A}_{2}\\ \;+8.50{12A}_{3}-164.{8187A}_{4}+155.{663A}_{5}\\ \;+0.352060A_{6}+10.{7823A}_{7}-18.{4979A}_{8}-76.90{59A}_{9}\\ \;=C_{\mathrm{PP}} \end{array}$$

(14) $$\begin{array}{l} 46.{8593A}_{1}-9.400{4A}_{2}+1.{83859A}_{3}-45.{8447A}_{4}\\ \;+1.{58366A}_{5}+0.0730{15A}_{6}+2.{31966A}_{7}\\ \;+43.{2512A}_{8}-16.{6275A}_{9}\\ \;=C_{\mathrm{CH}} \end{array}$$

(15) $$\begin{array}{l} 657.0{75A}_{1}+560.{177A}_{2}-107.{727A}_{3}-4826.0{9A}_{4}\\ \;+4745.{17A}_{5}-3.80{66A}_{6}-125.{563A}_{7}-1871.{61A}_{8}\\ \;+988.{299A}_{9}\\ \;=C_{\mathrm{PS}} \end{array}$$

where, C_PA_, C_MP_, C_CH_, C_PP_ and C_PS_ are the concentration of PA, MP, CH, PP and PS, respectively. The absorbance values at nine points, (230–270 nm) as in Figure 1 for samples were introduced to into the above equations. The concentration of the five component mixtures in Decamol Flu syrup was calculated.

Beer’s law was obeyed in the concentration 0–25.6, 0–15.0, 0–15.0, 0–45.0, 0–100.0 μg mL^-1^ for PA, MP, PP, CH and PS, respectively. Table 5 summarizes the linear regression parameters for the simultaneous determination of PA, MP, PP, CH and PS in their mixtures and limit of detections.

**Table 5:** Analytical Characteristics for analysis of PA, MP, PP, CH and PS by ILS method

**Analyte**	**Calibration equations**	**R**^**2**^	**Linear concentration range (μg mL**^**-1**^**)**	**LOD (μg mL**^**-1**^**)**
PA	Eq. (11)	0.9999	0-25.6	0.08
MP	Eq. (12)	0.9992	0-15.0	0.04
PP	Eq. (13)	0.9985	0-15.0	0.03
CH	Eq. (14)	0.9981	0-45.0	0.05
PS	Eq. (15)	0.9971	0-100.0	0.06

To check the reproducibility of the method, the R.S.D for five replicate determinations of 5.0 μg mL^-1^ each of PA, MP, PP, CH and PS, in the mixtures were obtained as 1.72, 2.13, 1.45, 2.02, and 1.33%, respectively. The mean recoveries were 99.95, 100.38, 100.64, 100.10 and 99.91% for PA, MP, PP, CH and PS respectively.

In order to obtain the accuracy and precision of the ILS method, several synthetic mixtures with different concentration ratios of PA, MP, PP, CH and PS were analyzed using the proposed method. The results are given in Table 6. The standard error of prediction (S.E.P) was calculated as [26]:

$$S.E.P.={\left(\frac{\sum_{j=1}^{N}{\left({\hat{C}}_{j}-C_{j}\right)}^{2}}{N}\right)}^{\frac{1}{2}}\;$$

where N is number of samples, *C*_*j*_ the concentration of component in jth mixture and *Ĉ*_*j*_ the estimated concentration. The standard error of calibration denoted by S.E.C is calculated as follows:

$$S.E.C.={\left(\frac{\sum_{j=1}^{N}{\left({\hat{C}}_{j}-C_{j}\right)}^{2}}{N-P-1}\right)}^{\frac{1}{2}}\;$$

where p is the number of analytes in the sample. Table 6 also shows the standard error of prediction and the standard error of calibration.

**Table 6:** Results for several experiments of validation tests for analysis of PA, MP, PP, CH and PS by ILS method

**Taken (μg mL**^**-1**^**)**				**Found (μg mL**^**-1**^**)**			**Recovery, %**							
**PA**	**MP**	**PP**	**CH**	**PS**	**PA**	**MP**	**PP**	**CH**	**PS**	**PA**	**MP**	**PP**	**CH**	**PS**
20.0	0.80	0.16	5.00	2.40	20.11	0.79	0.165	4.93	2.36	100.55	98.75	103.03	98.6	98.33
15.0	2.00	4.00	2.00	5.00	15.22	2.04	4.08	1.98	5.02	101.74	102.0	102.0	99.0	100.4
20.0	1.00	2.00	0.16	5.00	19.96	0.99	2.03	0.156	5.07	99.80	99.0	101.5	97.5	101.4
25.6	1.00	2.00	5.00	0.00	25.09	1.00	2.00	5.05	0.00	97.73	100.0	100.0	101.0	-
15.0	1.00	2.00	4.00	7.00	14.98	0.98	2.06	4.08	7.08	99.97	98.0	103.0	102.0	101.14
15.0	0.80	0.16	0.16	5.00	15.13	0.81	0.157	0.160	5.00	100.87	101.25	98.13	100.0	100.0
5.00	1.50	3.00	5.00	10.0	4.930	1.56	3.02	5.04	9.81	98.60	104.0	97.33	100.8	98.10
20.0	0.80	0.16	0.16	10.0	20.12	0.80	0.160	0.163	10.0	100.6	100.0	100.0	101.88	100.0
Mean recovery										99.95	100.38	100.64	100.1	99.91
S.E.P										0.233	0.127	0.1465	0.144	0.178
S.E.C										0.466	0.154	0.193	0.189	0.257

Good coincidence was observed for the assay results by applications of the two methods described in this paper. Comparison of the results in Table 4 and Table 6 proves that the analytical characteristics obtained by MCR method were similar to those obtained by ILS method. These methods appear to be the preeminant approach for almost quantitative analysis, since no knowledge for the sample composition is required beyond the concentrations of the constituents of interest, where the concentration of the analytes in real samples is always unknown.

### Analysis of pharmaceutical syrup

The proposed MCR and ILS methods were applied to the simultaneous determinations of PA, MP, PP, CH and PS in commercial syrup. Five replicates measurements were made for the determinations of PA, MP, PP, CH and PS. Satisfactory results were obtained for each compound in good agreement with claimed labels (Table 7). The results of the developed MCR method were compared with those of the proposed ILS method. Statistical comparison between the results was preformed with regards to accuracy and precision using t-test and F-ratio at 95% confidence limit (Table 7). There is no significant difference between the results of MCR and ILS methods.

**Table 7:** Determination of PA, MP, PP, CH and PS in commercial syrup using the proposed methods

**Sample No.**	**Concentration (μg mL**^**-1**^**)**					**Recovery, %**									
						**MCR**					**ILS**				
	**PA**	**MP**	**PP**	**CH**	**PS**	**PA**	**MP**	**PP**	**CH**	**PS**	**PA**	**MP**	**PP**	**CH**	**PS**
1	15.36	0.48	0.180	0.100	1.44	98.96	98.00	103.0	98.20	102.0	100.2	97.80	102.1	101.2	99.80
2	17.92	0.56	0.112	0.112	1.68	99.00	99.00	99.30	99.80	101.4	100.6	99.60	103.1	100.5	99.60
3	20.43	0.64	0.960	0.960	1.92	101.10	99.60	98.80	100.8	100.1	101.6	101.2	101.4	101.9	99.90
4	23.04	0.72	0.144	0.144	2.16	100.51	100.3	100.2	99.40	99.40	99.30	101.4	101.3	101.3	98.60
5	25.60	0.80	0.160	0.160	2.40	100.31	98.20	100.6	98.30	98.30	99.60	98.60	102.1	100.6	100.2
Mean recovery						100.0	99.00	100.4	99.30	100.6	100.3	99.70	102.0	101.1	99.60
S.D.^a^						0.970	0.960	1.620	1.090	0.990	0.900	1.580	0.720	0.570	0.610
t^b^						1.280	0.860	0.980	1.610	1.110					
F^b^						1.160	0.370	5.060	3.660	2.630					

^a^Standard deviation.

^b^Theoretical values for t and F at *p* =0.05 are 2.31 and 6.39, respectively.

## Conclusion

MCR and ILS developed methods were applied for the determination of five-component mixture of PA, MP, PP, CH and PA, where no knowledge for the sample composition is required beyond the concentrations of the constituents of interest. A comparative study of the use of MCR and ILS methods for the resolution of five-component mixture of PA, MP , PP, CH and PS has been accomplished showing that the two multivariate calibration methods provide, with adequate software support, a clear example of the high resolving power of these techniques. These methods have the advantage of high sensitivity, extremely low detection limit, good selectivity, rapid analysis and inexpensive instruments. Furthermore, while working with these methods, one does not need to use toxic organic solvents. In other words, they belong to green chemistry. The developed MCR and ILS methods were found to be suitable for the routine simultaneous determination of PA, MP, PP, CH and PS in pharmaceutical syrup.

## Competing interests

The authors declare that they have no competing interests.

## Authors’ contributions

MI designed the proposed method. RN planned and supervised the work. AS coordinated the study and drafting the manuscript. MI and RN analyzed the data statistically and revised manuscript critically. RN, MI and NS carried out the experimental work. All authors read and approved the final manuscript.
